# The Impact of COVID-19-Induced Changes at Schools on Elementary Students' School Engagement

**DOI:** 10.3389/fpsyg.2021.687611

**Published:** 2021-09-09

**Authors:** Kjærsti Thorsteinsen, Elizabeth J. Parks-Stamm, Marte Olsen, Marie Kvalø, Sarah E. Martiny

**Affiliations:** ^1^Department of Psychology, UiT The Arctic University of Norway, Tromsø, Norway; ^2^Department of Psychology, University of Southern Maine, Portland, ME, United States

**Keywords:** COVID-19 pandemic, school engagement, structural changes, elementary schoolchildren, well-being

## Abstract

In spring 2020, the COVID-19 pandemic led to the shutdown of schools in many countries. Emerging research documents the negative effects of the pandemic and particularly of the shutdown of schools on children's well-being. The present research extends this research by investigating how structural changes made in schools upon reopening to align with COVID-19 restrictions were related to children's emotional school engagement and subjective well-being. An online questionnaire with elementary school children and their parents conducted in Norway in June 2020 (*N* = 93 parent–child dyads; 46 boys, 47 girls; mean age children = 9.70 years, *SD* = 1.81) assessed structural changes in schools and children's coping with these changes, emotional school engagement, subjective well-being, self-reported performance in school, and demographics. Results showed that neither receiving a new teacher nor being assigned to a new (smaller) group were associated with negative outcomes. However, children who did not like their new group showed reduced emotional school engagement and subjective well-being, indicating that specific students particularly suffered from the pandemic-induced restrictions. The relationship between liking one's group and SWB was mediated by emotional school engagement. Applied and theoretical implications are discussed.

## Introduction

The emergence of the COVID-19 virus in China in late 2019 was the starting point of a major international health crisis. To restrict the spread of the virus, many countries implemented strict and far-reaching policy regulations including the shutdown of daycare centers and schools. By April 2020, 192 countries had closed their schools, affecting nearly 90% of the world's student population (UNESCO, 2020, as reported by Donohue and Miller, 2020). Depending on the infection rates within countries, the shutdown of schools and daycare centers lasted from several weeks to months, a disruption to students' education that experts have warned could have costly long-term consequences (e.g., Donohue and Miller, 2020; Fore, 2020; Golberstein et al., 2020; Prime et al., 2020). Upon reopening, schools in many countries implemented structural changes in line with strict disease prevention protocols including splitting classes into smaller groups and distancing.

In the present work, we explore how the structural changes implemented by Norwegian schools when reopening in late April 2020 affected elementary school children, focusing on two important outcome variables: children's attitudes towards school (i.e., emotional school engagement) and their general well-being. School engagement refers to students' commitment, involvement, and participation at school (Fredricks et al., 2004) and is associated with crucial outcomes such as achievement, academic resilience, and dropout rates (for a review see Fredricks et al., 2004). Therefore, school engagement is important to assess given the extensive changes to the school environment following the COVID-19 virus outbreak. Although emerging research shows that children's well-being suffered during the lockdown of society due to the pandemic (e.g., Hoffman and Miller, 2020; Spinelli et al., 2020; Xie et al., 2020; Martiny et al., 2021), little is known about how structural changes made to schools at reopening affected children's well-being. The present study, thus, makes an important contribution to our knowledge of the negative consequences of the pandemic on children.

### School Engagement

School engagement includes processes that promote learning and achievement and can be understood as the positive motivational force that ties students to schools (Ladd and Dinella, 2009). School engagement is both malleable and multifaceted (Fredricks et al., 2004). This means that students develop school engagement based on an interaction of individual characteristics and features of the environment, including family, community, culture, and the educational context (Fredricks et al., 2004). Secondly, it contains three components: (1) behavioral engagement, (2) cognitive engagement, and (3) emotional engagement (Fredricks et al., 2004). Behavioral engagement refers to following rules and adhering to norms at school, being involved in academic tasks, and participating in school-related activities. Cognitive engagement is defined as “psychological investment in learning, a desire to go beyond the requirements, and a preference for challenge” (Wehlage et al., 1989 as reported by Fredricks et al., 2004, pp. 63–64). Finally, emotional engagement refers to students' emotional reactions towards school (e.g., enjoyment, liking of school) and thus the emotional connections that tie students to school (Fredricks et al., 2004). Although all three dimensions are correlated among many students, other patterns are observed (e.g., students high in emotional engagement and low cognitive and behavioral engagement; Fredricks, 2011), and thus should be considered independently. Emotional engagement is the focus of the present study as it reflects the child's positive and negative reactions to the school experience (Fredricks, 2011).

Although many studies do not separately measure the individual components of school engagement (Upadyaya and Salmela-Aro, 2013), emotional engagement has been identified as a variable of interest as research suggest it is both directly (Valiente et al., 2007; Li and Lerner, 2011) and indirectly (Ladd et al., 2000; Li et al., 2010) related to academic outcomes (e.g., academic competence), dropping out of school (Fredricks et al., 2004), and well-being (Upadyaya and Salmela-Aro, 2013). Emotional engagement can fluctuate over time (Ladd and Dinella, 2009) and is influenced by features of the educational context like school size, teacher support, and peer acceptance (Fredricks et al., 2004; Li et al., 2010) as well as the home environment (e.g., maternal warmth; Li et al., 2010). The developmental trajectory of emotional engagement (e.g., if it decreases over time or remains high) significantly predicts important outcomes like grades, substance use, delinquency, and depression (Li and Lerner, 2011).

#### Antecedents of School Engagement: Environmental Factors

Given the malleability of engagement, in the present work, we are particularly interested in the impact of children's classroom environment. Research has shown that the classroom environment, including teachers and interactions with peers, are important determinants of students' school engagement and motivation at school (Ryan and Patrick, 2001), as well as sense of belonging at school (Goodenow, 1993; Skinner and Belmont, 1993). Research further shows that having negative relationships with peers predicts later maladjustment such as dropping out of school (for a review see Parker and Asher, 1987), and positive relationships with peers are positively related to students' school involvement (Berndt and Keefe, 1995) and academic engagement (Guthrie et al., 1995). Thus, earlier research shows that students' relationships with their teachers and peers can have consequences for their engagement in and motivation at school. Therefore, in the present work, we explore the role of both teachers and peers on elementary school children's emotional school engagement by examining how the changes involving grouping children into smaller groups and assigning groups to new teachers (implemented to reduce the spread of the COVID-19 virus in schools in spring 2020) was related to Norwegian elementary children's emotional school engagement.

#### Consequences of School Engagement: Academic Achievement and Continuance

In the past, research has investigated the achievement-related consequences of the three forms of school engagement (i.e., behavioral, cognitive, and emotional). Research focusing on behavioral and cognitive school engagement has consistently shown positive relationships between engagement and students' achievement and negative relationships with dropping out (Fincham et al., 1989; Skinner et al., 1990; Alexander et al., 1993; Fredricks et al., 2004; Ladd and Dinella, 2009; Lei et al., 2018). Much less is known about the role of emotional school engagement. Some earlier research that combined components of emotional and behavioral engagement showed positive relationships with achievement (e.g., Skinner et al., 1990), but it remains unclear whether emotional or behavioral engagement drives the effect. In addition, earlier research has shown correlations between identification with school (an aspect of emotional school engagement; Fredricks et al., 2004) and performance (Voelkl, 1997). More recently, researchers have used a more clear-cut and narrow definition of emotional school engagement by focusing on students' emotional connection to school. This research shows that students' emotional school engagement is positively related to classroom participation and academic achievement (Ladd et al., 2000; Lei et al., 2018). For example, a longitudinal study with 383 children (Ladd and Dinella, 2009) showed that early emotional school engagement predicts students' long-term academic growth. Taken together, research shows consistent links between behavioral and cognitive school engagement and students' performance and school continuance, and emerging evidence of positive long-term effects of young students' emotional school engagement. Therefore, in this work we focus on emotional school engagement as an understudied but potentially important concept both as an outcome of structural changes at schools and as a predictor of school performance.

### Children's Well-Being

Researchers around the world have documented decreases in children's well-being during the COVID-19 pandemic. In a national survey in the United States, 14% of parents reported a reduction in their children's behavioral health due to the pandemic (Patrick et al., 2020). Two studies from China showed an increase in children's symptoms of depression and anxiety (Duan et al., 2020; Xie et al., 2020). A study from Italy showed that parents' stress during the crisis had a negative impact on children's behavioral and emotional problems (Spinelli et al., 2020). Even in Norway, which experienced a relatively low number of cases and deaths in the first wave of the COVID-19 pandemic in spring 2020, children reported significant costs in well-being when schools were closed during the lockdown (Martiny et al., 2021). Researchers have argued that school closures played a significant role in this decrease in well-being (Hoffman and Miller, 2020), but the impact of the structural changes made to schools to align with COVID-19 restrictions upon reopening have not yet been examined.

#### Relationships Between School Engagement and Well-Being

The two general outcomes we are interested in—school engagement and well-being—have been linked in past studies. Zhu et al. (2019), for example, showed reciprocal relationships between elementary school children's subjective well-being (SWB) and behavioral school engagement; Datu and King (2018) found a reciprocal relationship between SWB and academic engagement in Filipino high school students. Other studies have looked at school engagement as a mediator between structural factors in school (e.g., specialized vs. regular classes; Orkibi and Tuaf, 2017) and SWB, and between mastery goal orientation and SWB (Yi et al., 2019). Liking school, as a component of students' affective engagement, is an important contributor to children's well-being (Baker and Maupin, 2009; but see Bradshaw et al., 2013).

### Structural Changes in Norwegian Schools due to the COVID-19 Pandemic

Elementary schools in Norway include grades 1 to 7. After closing all schools on March 12th, 2020, elementary schools reopened for the younger grades on April 27th (1st−4th grade) and May 11th for 5th−7th grade. At the time of reopening, all classes that contained more than 15 students (for 1st−4th grade) or 20 students (for 5th−7th grade) were split into smaller groups (with their original or new teachers). Practically, schools achieved this by redistributing students in the same year to the minimum number of groups necessary within the guidelines, thus all new groups were relatively similar in size, with 8–15 students per group in the lower grades and 10–20 students in the higher grades. Schools were required to keep the groups separate from each other (e.g., by assigning them to separate rooms and specific areas on the playground) and to maintain one-meter distance between students. Children stayed in the smaller groups for 5 (1st−4th grade) or 3 (5th−7th grade) weeks, until June 2nd. Then, the schools were allowed to go back to their normal classroom structure, such that children returned to their regular classes and teachers, but other restrictions such as distancing and good hand hygiene were still in place.

### The Present Research

We conducted an online questionnaire for elementary school children and their parents in Norway between June 8th and June 29th, between 6 and 26 days (*M*_*days*_ = 14) after the children returned to their regular classes. This means that by the time the questionnaire took place, children were back in their original (regular-sized) classes for an average of 2 weeks. With this questionnaire, we tested whether the temporary structural changes implemented in Norwegian elementary schools when schools reopened after the spring lockdown 2020 were associated with elementary school children's emotional school engagement and subjective well-being reported by both parents and children. We tested the effects of two structural changes on children's emotional school engagement and subjective well-being: (1) being taught by a new teacher and (2) being assigned to a smaller peer group. Next, we tested whether these structural changes had particularly detrimental effects for children who did not like their assigned group. We also explored whether children who reported not liking their new peer group had also shown lower emotional school engagement before the pandemic using retrospective reports from parents. Then we tested whether the relationship between dissatisfaction with the assigned small group and SWB was mediated by emotional school engagement. In addition, as past research has demonstrated that, as an environmental factor, family structure (e.g., single vs. two-parent household) predicts students' school engagement (e.g., through distance regulation and family resources; Bartle-Haring et al., 2012; Havermans et al., 2014), we also explored whether family structure was related to children's emotional school engagement as reported by parents. Finally, we investigated the relationship between emotional school engagement and children's performance and whether the effects of structural changes on performance were mediated by emotional school engagement.

## Materials and Methods

The study was approved by the Norwegian Center for Research Data and the Department of Psychology's at UiT The Arctic University of Norway's board for research ethics before data collection began.

### Participants

The inclusion criterion for the parent sample of the present study was being a parent of an elementary school child in Norway. The inclusion criterion for the children sample was being an elementary school child in Norway whose parents had answered the parent questionnaire. This study was part of a larger research project investigating parents' and children's well-being during the COVID-19 pandemic (Martiny et al., 2021; Thorsteinsen et al., 2021).

273 elementary school parents and 98 (35.9%) of their elementary school children answered an online questionnaire about school engagement and family well-being after the first outbreak and lockdown due to the COVID-19 pandemic in Norway. To strengthen our design, we asked both parents and children to report on the child's experience. This method allowed us not only to investigate the consistency between the two sources, but also the robustness of our findings (i.e., by running the same analyses with the measures from different sources). Only families in which both one parent and one child completed the questionnaire were included in the present analyses. We excluded three of the 98 parent–child dyads because we were unable to pair the children's questionnaires to their parents' from the self-generated codes. We also excluded two dyads because the children indicated at the end of the questionnaire that they did not understand the questions. The final sample of 93 parent–child dyads had an equal gender distribution for children with 46 boys and 47 girls, but was unbalanced in terms of parents' gender with mothers making up the majority of respondents (*n* = 87). The mean age for the children was 9.70 years (*SD* = 1.81, range 6 years and 5 months−13 years and 3 months) and the mean age of the parents was 39.98 years (*SD* = 6.23, range 26–60 years). Twenty children lived in a single-parent home, whereas 73 children lived in a two-parent home. The median and mode income category for parents in the sample was between NOK 460,000 and NOK 1,200,000; 40 participants reported a lower income and two participants reported earning more. Parents worked a mean of 32.30 (*SD* = 13.80) hours a week and 33 of the parents were classified as being essential workers^1^. One of the parents in the sample was in a same-sex relationship. Three children and ten parents were not born in Norway. Correlation and descriptive statistics for the study variables and additional demographic variables can be found in the Supplemental Materials.

### Procedure

Invitations were sent to 266 elementary schools across Norway: 40 principals confirmed that they would send the invitation with a link to the study to parents at their school;^2^ 17 principals responded that their school would not participate, and the rest did not reply. We additionally distributed the invitation through social media^3^ (i.e., an ad on Facebook that targeted 25 to 55-year-old parents and specifically asked for parents of elementary school children). In the parent questionnaire, participants were invited to participate in the study if they had at least one child attending elementary school. When parents had more than one child in elementary school, they were asked to choose one child and report on this child throughout the whole questionnaire. Parents first read detailed information about the study and were asked to give consent for both themselves and their child before completing their questionnaire. Item order was randomized within each measure except for the KIDSCREEN measure.^4^ Parents spent on average 15 mins on the questionnaire. At the end of the questionnaire, parents could choose to either have their child complete their questionnaire immediately by being directly redirected to the children's questionnaire or later as they also received the link to the questionnaire *via* e-mail. Parents could participate in a lottery for five gift cards (NOK 500).

Parents were asked to provide their self-generated code in the beginning of the children's questionnaire and encouraged to help their child get started with the questions. They were also given instructions on how children could click on an audio button to have each page read to them (including instructions, items, and scale points) and were asked to be available while their child completed the questionnaire in case there were questions. Before answering the children's questionnaire, children received tailored information about the study and gave their consent by clicking on a consent button. We created a child-friendly online questionnaire by presenting instructions and items in a large font, using short and understandable wording, and presenting one item per page. Most scale points were illustrated with visual images (see Supplemental Materials). 53 children reported that they received help from an adult filling in the questionnaire; 40 children reported not receiving help from an adult. Children took on average 15 mins to complete the questionnaire.

### Measures

Complete scales can be found in the Supplemental Materials in the order presented; additional measures that were assessed in this study are reported in the Supplemental Materials. All information was presented in Norwegian.

#### Structural Changes at School

Three yes/no-questions were used to assess structural changes at the children's schools due to the COVID-19 restrictions and children's reactions. Parents were asked whether their children were assigned to a new teacher after the schools reopened. Children were reminded of potential changes in schools that some children had experienced after the reopening of school (e.g., being divided into smaller groups, having a new teacher, or having to change classrooms) before they were asked “Was your class divided into smaller groups?.” If they answered yes, they were asked “Did you want to switch groups in your class?.”

#### Emotional School Engagement

Children's emotional school engagement was measured using items from the School Liking and Avoidance Questionnaire (Ladd and Price, 1987). The questionnaire consists of two subscales—school liking and school avoidance—comprising nine and five items each. In order to keep the questionnaire length reasonable, we only included six items. Both parents and children answered the items about how the child felt about school after the schools had reopened (e.g., “Does your child/Do you like being in school?”) on a Likert scale from 1 (never) to 5 (always). In addition, parents were asked to report how the child felt about school before the pandemic (retrospectively). A factor analysis using unweighted least squares extraction and a PROMAX rotation (as the original two subscales correlate) showed that the six items loaded on one factor, but one of the items^5^ (i.e., “Do you feel happier when it's time to go home from school”) showed low communalities for both parents' and children's reports (*h*^2^ < 0.2) and lower factor loadings (< 0.4) for both parents' and children's reports. Reliability analyses showed that the Cronbach's alpha increased when this item was excluded. The scale variable was thus constructed without this item and showed good reliability (α_parents_ = 0.91 [at both time points]; α_children_ = 0.85).

#### Children's Well-Being

To assess children's subjective well-being, both parents and children completed the cross-culturally validated Norwegian version of the KIDSCREEN-10 index (translated by Haraldstad et al., 2006 as reported by Ravens-Sieberer and the European KIDSCREEN Group, 2006). The measure includes 10 items covering facets of children's well-being with reference to the last week^6^ (e.g., “Has your child/Have you felt fit and well?”). Each question is answered on a 1 (never/not at all) to 5 (always/extremely) point Likert scale. After recoding two items, higher values indicated more positive well-being and a Rasch-scaled single score was computed (see Ravens-Sieberer and the European KIDSCREEN Group, 2006 for the procedure). The resulting index can be compared to existing European norm data, with an approximate mean of 50 and standard deviation of 10 (Ravens-Sieberer and the European KIDSCREEN Group, 2006). The scale showed good reliability (α_parents_ = 0.81; α_children_ = 0.79).

#### School Performance

We measured performance in mathematics, Norwegian, and English with one item each. Children were asked to think about how they were doing in school and then rate how much they agreed/disagreed with the statement “I am doing well in math/Norwegian/English at the moment” on a scale from 1 (not at all) to 5 (extremely). The three items correlated significantly (see correlation table in Supplemental Materials), and a general school performance variable was created (α = 0.70).

## Results

The data and code can be found on Open Science Framework (link: osf.io/4frk2). Descriptive statistics and correlations between the dependent variables, predictors, and covariates (child gender and age) are presented in Table 1. An extended table with additional demographics is presented in the Supplemental Materials. In the results section we report the parents' responses. Analyses of children's self-reports show similar patterns and are summarized in the last paragraph (for detailed results see Supplemental Materials).

**Table 1:** Descriptive statistics and correlations.

**Variables**	***M***	***SD***	***n***	**1**.	**2**.	**3**.	**4**.	**5**.	**6**.	**7**.	**8**.	**9**.	**10**.	**11**.	**12**.
**Emotional school engagement**														
1. Parent-reported	3.87	0.88	93	1										
2. Parent-reported, retrospective	3.91	0.83	93	0.77^**^	1									
3. Child-reported	3.74	0.90	93	0.71^**^	0.74^**^	1								
**Well-being**														
4. Parent-reported	47.33	10.60	93	0.63^**^	0.52^**^	0.42^**^	1							
5. Child-reported	49.82	10.59	93	0.39^**^	0.49^**^	0.56^**^	0.47^**^	1						
**Other variables**														
6. New teacher after reopening	0.81	0.40	93	−0.04	0.07	0.06	0.01	0.02	1					
7. Class divided into smaller groups	0.35	0.48	93	0.00	0.08	−0.06	0.04	0.04	0.25^*^	1				
8. Wanting to switch groups	0.63	0.49	60	−0.28^*^	−0.16	−0.17	−0.31^*^	−0.14	−0.07	−	1			
9. School performance	3.77	0.92	93	0.36^**^	0.34	0.46^**^	0.23^*^	0.45^**^	−0.03	−0.07	−0.00	1		
10. Child age	9.70	1.81	93	0.04	−0.04	−0.11	−0.01	0.01	−0.13	−0.12	−0.11	−0.04	1	
11. Child gender	0.51	0.50	93	0.03	0.22^*^	0.13	−0.02	0.06	−0.02	0.21^*^	0.29^*^	0.13	−0.02	1
12. Family structure	0.78	0.41	93	0.29^**^	0.30^**^	0.36^**^	0.25^*^	0.33^**^	0.06	−.22^*^	−0.14	0.17	−0.04	−0*s*.05	1

*Having a new teacher, Class divided into smaller groups, and Wanting to switch groups are coded 1 for yes and 0 for no; Child gender is coded 0, boys; 1, girls; Family structure is coded 0, single-parent families and 1, two-parent families.*

** *Correlation is significant at the 0.01 level.*

* *Correlation is significant at the 0.05 level (2-tailed)*.

### Parents' Reports

#### Are Structural Changes at School Related to Children's Emotional School Engagement?

First, we tested whether the two structural changes at school (new group and new teacher) made in response to the COVID-19 pandemic related to children's emotional school engagement in a one-way analysis of covariance with child age and gender as covariates. Results showed that 18 children had a new teacher after the reopening, while 75 kept the same teacher as before the lockdown. The children who were taught by a new teacher (*M* = 3.81, *SD* = 1.02) did not significantly differ in emotional school engagement from the children taught by the same teacher (*M* = 3.89, *SD* = 0.85). Thus, students' emotional school engagement was not affected by having a new teacher, *F*_(1, 89)_ = 0.08, *p* = 0.778. Sixty children were assigned to smaller groups after schools reopened and 33 remained in their normal classes. Overall, this structural change was not associated with emotional school engagement, *F*_(1, 89)_ < 0.01, *p* = 0.990. The children who were divided into smaller groups (*M* = 3.87, *SD* = 0.96) did not report a lower level of emotional school engagement than those who remained in their normal class (*M* = 3.87, *SD* = 0.74) as reported by their parents.

Next, we tested whether being happy with the new peer group was related to children's emotional school engagement. Of the 60 children who were divided into smaller groups, 22 children reported that they had wanted to switch groups. These children reported significantly lower emotional school engagement (*M* = 3.53, *SD* = 0.95) than the 38 children who did not want to switch groups (*M* = 4.07, *SD* = 0.92), *F*_(1, 56)_ = 4.55, *p* = 0.037, $\eta_{p}^{2}$ = 0.08 (as reported by their parents).

The parents' questionnaire included items both on children's reopening emotional school engagement (reported above) and their emotional school engagement before the pandemic (asked retrospectively). Therefore, to further explore the relationship between satisfaction with the child's peer group and engagement, we conducted an exploratory analysis to test whether children who had reported not liking their new peer group had also shown lower emotional school engagement before the pandemic. Two-way repeated measures ANOVA showed no significant change in emotional school engagement from T1 to T2, *F*_(1, 58)_ = 2.28, *p* = 0.126, but the interaction between children's liking of their new group and time approached significance, *F*_(1, 58)_ = 2.92, *p* = 0.093. Simple slopes analyses revealed that children who did not like their new group descriptively reported lower emotional school engagement prior to the pandemic (*M* = 3.78, *SD* = 0.87) and this engagement then declined further after schools implemented the pandemic-related changes (*M* = 3.53, *SD* = 0.95), *F*_(1, 58)_ = 4.09, *p* = 0.048. Children who liked their group reported the same level of school engagement prior to the pandemic (*M* = 4.06, *SD* = 0.83) and after the reopening (*M* = 4.07, *SD* = 0.92), *F*_(1, 58)_ = 0.27, *p* = 0.870, indicating that these childrens' emotional school engagement did not change. These patterns remained when including age and gender as covariates, *F*_(1, 56)_ = 2.09, *p* = 0.154. None of the covariates influenced changes in emotional school engagement in this model, *ps* < 0.600.

#### Are Structural Changes at School Related to Children's Well-Being?

Using the same procedure as above, we tested the relationship between structural changes and children's subjective well-being. There were no differences in well-being between children taught by a new teacher (*M* = 47.63, *SD* = 10.44) and children taught by the same teacher (*M* = 47.26, *SD* = 10.71), *F*_(1, 89)_ = 0.01, *p* = 0.907. Children assigned to a new group (*M* = 47.68, *SD* = 11.18) reported the same level of subjective well-being as children staying in the same group (*M* = 46.71, *SD* = 9.60), *F*_(1, 89)_ = 0.21, *p* = 0.645. However, in line with the results on emotional school engagement, children who did not like the groups they were assigned to reported lower well-being (*M* = 43.17, *SD* = 9.53) than children who did not (*M* = 50.28, *SD* = 11.34), *F*_(1, 56)_ = 5.75, *p* = 0.020.

#### The Relationship Between Emotional School Engagement and Well-Being

As can be seen in Table 1, emotional school engagement was positively related to child well-being. This relationship remained stable when tested in a linear regression analysis controlling for child age and gender, *b* = 0.63, *t*_(89)_ = 7.63, *p* < 0.001, *f*^2^ = 0.65. We then explored whether the relationship between not liking one's group and well-being was mediated by emotional school engagement (Process model 4, Hayes, 2018, 50,000 bootstrap samples, see Table 2). Wanting to switch groups predicted emotional school engagement [*a* = −0.57 (−1.10; −0.04)], which in turn predicted well-being [*b* = 7.72 (5.45; 9.99)]. A bias corrected confidence interval for the indirect effect of switching groups [*ab* = −4.39 (−8.92; −0.10)] did not include zero, meaning that emotional school engagement mediated the relationship between wanting to switch groups and well-being.

**Table 2:** Mediation model for the relationship of wanting to switch groups and well-being *via* emotional school engagement (*n* = 60).

		**Emotional school engagement (M)**				**Well-being (Y)**		
		**Coeff. (LLCI; ULCI)**	***SE***	***p***		**Coeff. (LLCI; ULCI)**	***SE***	***p***
Wanting to switch groups (X)	a	−0.57 (−1.10; −0.04)	0.27	0.037	c'	−2.89 (−7.59; 1.81)	2.34	0.801
Emotional school engagement (M)					b	7.72 (5.45; 9.98)	1.13	<0.001
Gender		0.11 (−0.41; 0.62)	0.26	0.685		−1.57 (−5.97; 2.83)	2.20	0.478
Age		0.03 (−0.13; 0.18)	0.08	0.728		−1.30 (−2.60; 0.00)	0.65	0.050
Constant	I_M_	3.66 (2.02; 5.30)	0.82	<0.001	I_Y_	33.73 (17.54; 49.93)	8.08	<0.001
		*R*^2^ = 0.08				*R*^2^ = 0.53		
		*F*_(3, 56)_ = 1.66		0.185		*F*_(4, 55)_ = 15.18		<0.001

*Confidence intervals are displayed at the 95% level*.

#### Additional Predictors and Outcomes of Emotional School Engagement

##### Family Structure as a Predictor

In line with earlier studies, correlational analyses showed that family structure was related to emotional school engagement and this relationship remained significant controlling for age and gender, *F*_(1, 89)_ = 8.30, *p* = 0.005, $\eta_{p}^{2}$ = 0.09. Children from two-parent families reported higher emotional school engagement (*M* = 4.01, *SD* = 0.77) than children from single-parent families (*M* = 3.39, *SD* = 1.09).

However, the patterns of results in the repeated measure ANOVA of parent-reported emotional school engagement (presented above) did not change when including family structure as a covariate. Furthermore, family structure did not influence changes in emotional school engagement from before the pandemic to the time of reopening, *F*_(1, 55)_ = 0.49, *p* = 0.487. We therefore did not include it as a covariate in the subsequent analyses.

##### School Performance as an Outcome

In line with earlier research, we found that children's emotional school engagement was correlated with their school performance and this relationship remained stable when controlling for age and gender, *b* = 0.36, *t*_(89)_ = 3.68, *p* < 0.001, *f*^2^ = 0.18. Therefore, we explored whether wanting to switch groups negatively affected children's school performance^7^ *via* emotional school engagement (Process model 4, Hayes, 2018, 50,000 bootstrap samples, see Table 3). Wanting to switch groups predicted children's emotional school engagement [*a* = −0.57 (−1.10; −0.04)], which in turn predicted children's school performance [*b* = 0.48 (0.24; 0.74)]. There was no direct relationship between wanting to switch groups and school performance, but a bias corrected confidence interval for the indirect effect [*ab* = −0.28 (−0.72; −0.00)] of switching groups did not include zero, meaning that emotional school engagement mediated the relationship.

**Table 3:** Mediation model for the relationship of wanting to switch groups and school performance via emotional school engagement (*n* = 60).

		**Emotional school engagement (M)**				**School performance (Y)**		
		**Coeff. (LLCI; ULCI)**	***SE***	***p***		**Coeff. (LLCI; ULCI)**	***SE***	***p***
Wanting to switch groups (X)	a	−0.57 (−1.10; −0.04)	0.27	0.037	c'	−0.09 (−0.42; 0.60)	0.26	0.736
Emotional school engagement (M)					b	0.48 (0.24; 0.74)	0.13	<0.001
Gender		0.11 (−0.41; 0.63)	0.26	0.685		−0.55 (0.62; 1.03)	0.24	0.028
Age		0.03 (−0.13; 0.18)	0.08	0.728		−0.02 (−0.26; 0.13)	0.07	0.795
Constant	I_M_	3.66 (2.02; 5.30)	0.82	<0.001	I_Y_	1.12 (−0.66; 2.90)	0.89	0.212
		*R*^2^ = 0.08				*R*^2^ = 0.28		
		*F*_(3, 56)_ = 1.66		0.185		*F*_(4, 55)_ = 5.32		0.001

*Confidence intervals are displayed at the 95% level*.

### Children's Self-Reports

Parents' and children's reports correlated strongly for emotional school engagement and moderately for well-being (see Table 1). In general, the results for children's self-reported emotional school engagement and well-being showed similar, but partly non-significant, patterns in the same direction as the results reported by the parents. Similar to the results with the parents' report, being assigned to a new group or a new teacher were not associated with lower self-reported emotional school engagement or well-being. Descriptively, children who wanted to switch groups reported lower emotional school engagement and well-being than children who did not. Finally, their emotional school engagement was significantly related to well-being, family structure and school performance, also when controlling for covariates (see Table 1 and the Supplemental Materials).

## Discussion

Results of the present study show that whether or not elementary students were temporarily assigned to a new teacher or a new peer group upon their return to school was not necessarily associated with decreases in their emotional school engagement or their SWB. However, the children who did not like their smaller peer group reported reduced emotional school engagement and subjective well-being even after they were back in their regular classes for approximately 2 weeks. In addition, we found that the relationship between liking one's group and subjective well-being was mediated by emotional school engagement. This is in line with earlier work showing school engagement as a mediator between structural factors in school and SWB (e.g., Orkibi and Tuaf, 2017), and the importance of peer acceptance for emotional engagement in school (e.g., Fredricks et al., 2004). In line with past findings (e.g., Bartle-Haring et al., 2012; Havermans et al., 2014), children living in single-parent (vs. two-parent) households also reported lower school engagement after the reopening of schools. However, change in school engagement from before the pandemic (asked retrospectively) to the time of reopening was not influenced by family structure. Instead, the change was associated with not liking one's group. Finally, we found that emotional school engagement was related to children's self-reported performance and mediated the link between not liking one's peer group and performance.

The present study makes an important contribution not only to existing literature on the antecedents and consequences of children's school engagement and well-being under normal circumstances (Fredricks et al., 2004; Upadyaya and Salmela-Aro, 2013), but also to the emerging literature that focuses on the negative consequences of the COVID-19 pandemic on children and parents (Hoffman and Miller, 2020; Spinelli et al., 2020; Martiny et al., 2021). We extend previous work on the effects of lockdowns and school closures on children by examining features of a previously unexplored context: classrooms altered by COVID-related restrictions. By integrating empirical evidence related to school engagement, well-being, and the effects of the pandemic on schoolchildren, the present work demonstrates that specific antecedents of well-being and emotional school engagement continue to play a role during a worldwide health-related crisis, and introduces new insights into factors associated with particularly negative responses to pandemic-related restrictions in schools.

Taken together these results have important implications. Children who were placed into small groups that they did not like reported reduced emotional school engagement even when they were back in their original classes. From an applied perspective, these findings highlight the importance of student placement more broadly. Past research has shown that few administrators use a truly random assignment of students to classrooms, often attempting to “balance” classes by relying on impressions of students' abilities, personalities, learning styles, and potential compatibility with teachers (Paufler and Amrein-Beardsley, 2014). In times of crisis, the present research suggests placing children into small peer groups they are unhappy with can have negative effects on their emotional school engagement that in turn can have consequences for their well-being and performance. These findings also merit attention from a research perspective, as they show that it is important to adopt a differentiated perspective when investigating children's school engagement, well-being, and performance, not unlike research examining outcomes for individuals grouped by their developmental trajectories of engagement (Li and Lerner, 2011). Looking merely at averages underestimates the negative effects experienced by certain individuals.

Further, the pandemic led to mid-year placements into new peer groups that occurred without the normal concomitant changes (e.g., a new school year, grade, performance expectations). Due to the pandemic-related restrictions, classes were divided (or not) purely based on a structural characteristic, namely whether they contained more than 15 students. Focusing on the students who were affected by this change and comparing those who were not happy with the change with those who were, gives us the unique opportunity to compare a target group with a natural control group. The effects of group placement, unadulterated by other concomitant factors, allowed us to focus on the relationships between structural changes in classrooms (and students' satisfaction with these changes) and students' engagement, well-being, and school performance. That dissatisfaction with one's group in these conditions is associated with such negative effects in important outcomes highlights the need to consider peer groups in placement decisions not only in times of crisis but also within regular school routines.

Our findings also suggest that it is important for schools to identify the children who would be most at risk of feeling out of place in school. Children in our sample who were uncomfortable in the new groups reported descriptively lower emotional school engagement prior to the pandemic, and this engagement then declined further when schools implemented the pandemic-related changes. Thus, it appears that the most vulnerable children suffered most from the changes made in school in response to the ongoing pandemic. This finding is in line with earlier research demonstrating that the pandemic had the most detrimental effects on at-risk young children (Dooley et al., 2020; Martin and Sorensen, 2020). Thus, the present work makes an important contribution to existing literature by highlighting specific ways that vulnerable children suffered during the pandemic, even when schools reopened. In addition to these pandemic-specific effects, the present findings highlight the need for more research under normal circumstances on the importance of placing children—particularly high-risk children—into peer groups they are happy about in school, to support their emotional engagement and feelings of belonging.

### Limitations and Outlook

In the present study, we had limited time and opportunity to assess additional variables. For example, other mental, psychological, and behavioral factors may have impacted students' well-being, performance, and adjustment during the pandemic, and it is possible that these could serve as confounding variables. In addition, we did not measure the exact size of the smaller groups students were assigned to during the first stage of reopening of schools as we did not want to overwhelm the children and parents with our survey. However, our analyses focused mostly on students who came from a class with more than 15 (1st−4th grade) or 20 (5th−7th grade) students and consequently were divided into groups with a minimum of 8–15 students and 10–20 students, respectively.

In the present study, we used a cross-sectional design that does not allow any causal interpretation. Further research should investigate causal links between structural changes at school, emotional school engagement, well-being, and performance, as well as potential mediators, by using longitudinal or experimental designs to allow for causal conclusions. The sample size in the present research is relatively small and future research should replicate the present findings with larger sample sizes. In addition, the data were collected online. This is associated with both benefits and limitations (e.g., Heiervang and Goodman, 2011). One limitation of online studies is that error variance is increased since children answered the questionnaire at home and not under controlled conditions in the lab. In addition, there is the potential risk of oversampling students from higher social classes while reaching fewer students with non-traditional backgrounds such as immigrant students and students from families with low socio-economic status. Although selective participation from online questionnaires can affect point estimates, patterns of associations are more robust to this threat (e.g., Heiervang and Goodman, 2011). Furthermore, in the present work, we asked both parents and children to report on the child's experience. This method allowed us not only to investigate the consistency between the two sources, but also the robustness of our findings (i.e., by running the same analyses with the measures from different sources). We found stronger and more consistent patterns with the parents' data than with the children's data, which might be due to the problem mentioned above namely that some children—especially the younger ones—might have had difficulties filling out the online questionnaire. However, the parents' reports and the children's self-reports were highly correlated and we found similar patterns in both data sets, confirming the robustness of the present results and the validity of parental reports for child-related measures. Finally, school performance was assessed subjectively, by asking children how well they are doing in three main subjects (mathematics, Norwegian, and English). As children in Norwegian elementary schools do not receive grades (i.e., only verbal feedback), children may not accurately judge their performance.

## Conclusion

The present work investigated how structural changes implemented in Norwegian elementary schools to align with COVID-19 restrictions were associated with children's emotional school engagement and subjective well-being. We found that children who were unhappy with the new group they were assigned to showed lower general well-being and emotional school engagement. Reduced emotional school engagement not only mediated the relationship between being unhappy with their peer group and subjective well-being, but was also linked to lower performance. School authorities should consider these differential effects of structural changes at schools, particularly in times of crisis in which children appear to be particularly vulnerable.

## Data Availability Statement

The datasets generated for this study can be found in online repositories. The names of the repository/repositories and accession number(s) can be found below: The data and code can be found on Open Science Framework (osf.io/4frk2).

## Ethics Statement

The studies involving human participants were reviewed and approved by Department of Psychology's at UiT The Arctic University of Norway's board for research ethics. Written informed consent to participate in this study was provided by the participants' legal guardian/next of kin.

## Author Contributions

KT, MK, MO, and SEM contributed to conception and design of the study. MO and KT organized the data. KT and SEM performed the statistical analysis. EJP-S, SEM, and KT wrote sections of the manuscript. All authors contributed to manuscript revision, read, and approved the submitted version.

## Conflict of Interest

The authors declare that the research was conducted in the absence of any commercial or financial relationships that could be construed as a potential conflict of interest.

## Publisher's Note

All claims expressed in this article are solely those of the authors and do not necessarily represent those of their affiliated organizations, or those of the publisher, the editors and the reviewers. Any product that may be evaluated in this article, or claim that may be made by its manufacturer, is not guaranteed or endorsed by the publisher.

## Footnotes

^1^Participants reported occupation in open-ended questions coded as ‘Essential worker' or not as defined by the Norwegian government (https://www.regjeringen.no/no/tema/samfunnssikkerhet-og-beredskap/innsikt/liste-over-kritiske-samfunnsfunksjoner/id2695609/). The first 10% of the participants were categorized by Author 4 and 5 individually. They showed a high agreement (κ = 0.92); therefore Author 5 finished the categorization.

^2^All schools in Norway use digital communication with parents, e.g., e-mail or specialized software applications.

^3^At the time the Facebook ad was published, 62 participants of the final sample had already completed the questionnaire (i.e., recruited through their school). As we did not ask participants to indicate how they were recruited, we do not know how many of the remaining 31 participants were recruited via Facebook or school.

^4^Copyright prohibits randomization of KIDSCREEN-10.

^5^This item was challenging to translate and we later realized that the Norwegian translation has a slightly different meaning than the original English item.

^6^Two of the items refer to school in the official Norwegian translation (getting on well and being able to pay attention in school).

^7^When the performance items were analyzed separately, results were replicated with mathematics and Norwegian, whereas English performance showed the same pattern but did not reach the conventional significance level.
