# Supplementary material for: The Impact of COVID-19-Induced Changes at Schools on Elementary Students' School Engagement
Source: Front Psychol. 2021 Sep 9;12:687611. doi: 10.3389/fpsyg.2021.687611 (PMC8458819; doi:10.3389/fpsyg.2021.687611)
Supplement: Supplementary file 1 [file Data_Sheet_1.docx]

**Supplemental materials**

In this section, we report the following additional information: A) an extended correlation table including additional demographic variables that might be of interest, Table 1; B) the detailed results of the reported analyses using children’s self-report: C) a detailed overview of the original measures used in the study, Tables 2 - 4.

**A. Additional Demographic Variables**

Table 1

*Correlation and Descriptive Statistics for Study Variables and Additional Demographic Variables*

 *Note.* Having a new teacher, Class divided into smaller groups, and Wanting to switch groups are coded 1 for yes and 0 for no; Child gender and Parent gender are coded 0 for male and 1 for female; COVID-19 risk group, child and COVID-19 risk group, parent are coded 0 for yes and 1 for no; Family structure is coded 0 for single-parent families and 1 for two-parent families; Born in Norway, parent is coded 0 for yes and 1 for no; Income, parent has the categories 1 = NOK 0 - 320 000, 2 = NOK 320 000 - 460 000, 3 = NOK 460 000- 1 200 000, 4 =NOK 1 200 000 - 2 000 000, and 5 = above NOK 2 000 000; Essential worker, parent and COVID-19 risk group, parent was coded 0 for yes and 1 for no.

**Correlation is significant at the 0.01 level. *Correlation is significant at the 0.05 level (2-tailed).

**B. Results using the Children’s Self-Reports**

Below we report the detailed analyses of the results summarized in the manuscript under the paragraph titled “Children’s self-reports.” We conducted the same analyses as in the main results section, but instead of parents’ reports of how their children are doing, these are the results of analyses with children’s self-report of emotional school engagement and subjective well-being.

***Do Structural Changes at School Affect Emotional School Engagement?***

First, in a one-way analysis of covariance with child age and gender as covariates, we tested whether structural changes at school due to the Covid-19 pandemic influenced children’s emotional school engagement. Results showed that the children who were taught by a new teacher (*M* = 3.86, *SD* = 0.88) did not significantly differ on emotional school engagement from the children taught by the same teacher (*M* = 3.72, *SD* = 0.91). Thus, emotional school engagement was not affected by children having a new teacher, *F*(1, 89) = 0.23, *p* = .631. Emotional school engagement was also not affected by being divided into smaller groups, *F*(1, 89) = 1.05, *p* = .309, and children who were divided into smaller groups (*M* = 3.70, *SD* = 0.91) did not report a lower level of emotional school engagement than the children who remained in their normal class (*M* = 3.82, *SD* = 0.88).

Descriptively, we found that the 22 children who reported that they wanted to switch groups reported lower emotional school engagement (*M* = 3.50, *SD* = 0.98) than the 38 children who did not indicate that they wanted to switch groups (*M* = 3.82, *SD* = 0.86). But the difference was not statistically significant, *F*(1, 56) = 2.01, *p* = .162.

***Do Structural Changes at School Affect Children’s Well-Being?***

Likewise, there were no differences between children taught by a new teacher (*M* = 50.31, *SD* = 14.09) and children taught by the same teacher (*M* = 49.70, *SD* = 9.69) in well-being, *F*(1, 89) = 0.05, *p* = .817. Children assigned to a smaller group (*M* = 50.11, *SD* = 12.33) also reported the same level of subjective well-being as children staying in the same group (*M* = 49.29, *SD* = 6.47), *F*(1, 89) = 0.07, *p* = .800. In line with findings on emotional school engagement, children who wanted to switch groups similarly reported descriptively lower levels of well-being (*M* = 47.90, *SD* = 13.78) than children who did not want to switch groups (*M* = 51.39, *SD* = 11.40), but the effect for the children’s reports on well-being were also not significant, *F*(1, 56) = 1.35, *p* = .250.

***The Relationship between Emotional School Engagement and Well-Being***

As can be seen in Table 1 (of the Supplemental Materials), emotional school engagement was positively related to child well-being (*r* = .57, *p* < .001). This relationship remained stable when testing it in a linear regression analyses, *b* = .57, *t*(89) = 6.40, *p* < .001, *f^2^* = 0.47, controlling for child age and gender.

***Additional Predictor and Outcomes of Emotional School Engagement***

***Family Structure as a Predictor.*** Family structure was also related to emotional school engagement reported by the children (*r* = .36, *p* < .001). This relationship also remained significant when controlling for covariates (i.e., age and gender), *F*(1, 89) = 14.35, *p* < .001, *η_p_^2^* =.14. The 73 children from two-parent families reported more positive emotional school engagement (*M* = 3.91, *SD* = 0.73) than the 20 children from single-parent families (*M* = 3.12, *SD* = 1.12).

***School Performance as an Outcome.*** We further found that self-reported emotional school engagement also was correlated with their school performance and that this relationship remained stable when testing it in a linear regression analyses, *b* = .45, *t*(89) = 4.77, *p* < .001, *f^2^* = 0.28, controlling for child age and gender.

**C) Overview of Measures**

Table 2 to 4 include variables, visualization, and Norwegian and English wording of the original items. The original audio files (in Norwegian) can be found in the “Audio files”-folder under the OSF project folder. Please note that the KIDSCREEN-10 is copyrighted to the KIDSCREEN Group and therefore the items will not be reported. In order to receive and use the original scales of the KIDSCREEN-10, you may contact the KIDSCREEN group directly (https://www.kidscreen.org). In addition to the measures reported here, in the children’s questionnaire we assessed: general attitudes towards the Covid-19 pandemic, experiences of home schooling, positive and negative emotions, gender stereotypes and career aspirations, status of communal and agentic occupations, and demographics. In the parents’ questionnaire we assessed: retrospective measures of child well-being under normal circumstances and during the lockdown, parent well-being, stress, essentialist beliefs about parenthood, domestic responsibilities, attitudes towards equality between sexes, demographics about themselves, their child, and their partner, and at the end participants were given the opportunity to give additional information or comments.

***Parents***

Table 2

*The Six Items Included from School Liking and Avoidance Questionnaire to Measure Emotional School Engagement (Ladd & Price, 1987; items retrieved from Smith, 2011)*

**

***Children***

Table 3

*The Six Items Included from School Liking and Avoidance Questionnaire to Measure Emotional School Engagement (Ladd et al., 2000)*

Table 4

*School Performance Measure (OWN)*

| Language | Items | | | | | | |
| --- | --- | --- | --- | --- | --- | --- | --- |
|  | Instructions | Text | Scale points | | | | |
|  |  | Scale visualizations | 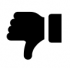 |  |  |  | 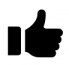   \|  \| \| --- \| |
| English | Think of how you are doing in the topic and choose the answer you feel fits you best. How are you doing in school? | I am doing well in mathematics at the moment. | Not at all | Slightly | Moderately | Very | Extremely |
|  |  | I am doing well in Norwegian at the moment. | Not at all | Slightly | Moderately | Very | Always |
|  |  | I am doing well in English at the moment. | Not at all | Slightly | Moderately | Very | Always |
| Norwegian | Tenk over hvordan du har gjort det i faget, og trykk på det svaret du synes passer best for deg. Hvordan gjør du det på skolen? | Jeg gjør det bra i matematikk for tiden. | Ikke i det hele tatt | Litt | Ganske | Veldig | I høy grad |
|  |  | Jeg gjør det bra i norsk for tiden. | Ikke i det hele tatt | Litt | Ganske | Veldig | I høy grad |
|  |  | Jeg gjør det bra i engelsk for tiden. | Ikke i det hele tatt | Litt | Ganske | Veldig | I høy grad |

**References**

Ladd, G.W. & Price, J.M. (1987). Predicting children’s social and school adjustment following the transition from preschool to kindergarten. *Child Development, 58*, 1168-1189. <https://doi.org/10.2307/1130613>

Ravens-Sieberer, U., & the European KIDSCREEN Group. (2006). *The KIDSCREEN questionnaires—Quality of life questionnaires for children and adolescents—Handbook*. Lengerich: Pabst Science Publisher.

Smith, J. (2011). *Measuring school engagement: A longitudinal evaluation of the school liking and avoidance questionnaire from kindergarten through sixth grade*. Arizona State University, AZ.

<http://repository.asu.edu/attachments/56844/content/Smith_asu_0010N_10812.pdf>
